# microRNA sequencing for biomarker detection in the diagnosis, classification and prognosis of Diffuse Large B Cell Lymphoma

**DOI:** 10.1038/s41598-023-39271-7

**Published:** 2023-07-27

**Authors:** Ane Larrabeiti-Etxebarria, Nerea Bilbao-Aldaiturriaga, Javier Arzuaga-Mendez, Maialen Martin-Arruti, Luca Cozzuto, Ayman Gaafar, Irune Ruiz-Diaz, Isabel Guerra, Idoia Martin-Guerrero, Elixabet Lopez-Lopez, Angela Gutierrez-Camino

**Affiliations:** 1 Department of Genetics, Physical Anthropology and Animal Physiology, Faculty of Science and Technology, University of the Basque Country, UPV/EHU, Leioa, Spain; 2 Department of Biochemistry and Molecular Biology, Faculty of Science and Technology, University of the Basque Country, UPV/EHU, Barrio Sarriena s/n, 48940 Leioa, Spain; 3 Pediatric Oncology Group, BioCruces Bizkaia Health Research Institute, Barakaldo, Spain; 4 Hematologic Neoplasm Group, BioCruces Bizkaia Health Research Institute, Barakaldo, Spain; 5 Pathology Department, Donostia University Hospital, San Sebastián, Spain; 6 Centre for Genomic Regulation (CRG), The Barcelona Institute of Science and Technology, Barcelona, Spain; 7 Pathology Department, Cruces University Hospital, Barakaldo, Spain; 8 Pathology Department, Araba University Hospital, Vitoria, Spain; 9 Division of Hematology-Oncology, CHU Sainte-Justine Research Center, Montreal, Canada

**Keywords:** Cancer, Genetics, Biomarkers, Oncology

## Abstract

Despite being considered a single disease, Diffuse Large B Cell Lymphoma (DLBCL) presents with variable backgrounds, which results in heterogeneous outcomes among patients, with 40% of them still having primary refractory disease or relapse. Thus, novel biomarkers are needed. In addition, multiple factors regarding its pathogenesis remain unclear. In this context, recent investigations point to the relevance of microRNAs (miRNAs) in cancer. However, regarding DLBCL, there is inconsistency in the data reported. Therefore, in this work, the main goals were to determine a miRNA set with utility as biomarkers for DLBCL diagnosis, classification, prognosis and treatment response, as well as to decipher the mechanism of action of deregulated miRNAs in the origin of the disease. We analyzed miRNA expression in a cohort of 78 DLBCL patients and 17 controls using small RNA sequencing and performed a miRNA-mRNA interaction network analysis. This way, we were able to define new miRNA expression signatures for diagnosis, classification, treatment response and prognosis, and we identified plausible mechanisms of action by which deregulated miRNAs could be involved in DLBCL pathogenesis. In summary, our study remarks that miRNAs could play an important role in DLBCL.

## Introduction

Diffuse Large B Cell Lymphoma (DLBCL) is an aggressive type of non-Hodgkin lymphoma that represents the most common lymphoid malignancy in adults^1^. Despite the great heterogeneity observed among patients with DLBCL in terms of morphology, genetics, and biological behavior, most of them are treated with standard chemotherapy regimens, which allow complete remission in 75–80% of patients^2,3^. Nevertheless, the remaining patients will be refractory to first-line chemotherapy, and 30–40% will relapse after obtaining a complete remission. Therefore, the identification of new biomarkers for a better management of DLBCL patients is an urgent priority.

In this context, microRNAs (miRNAs), as post-transcriptional regulators of gene expression and biological functions, have shown potential as diagnostic, classification and prognostic predictors in cancer, although differences among studies were identified. For instance, miR-150-5p was downregulated in DLBCL in four studies^4–7^, while showed upregulated expression in another study^8^ and unchanged in another one^9^. Similarly, miR-222-3p was associated with good prognosis in four studies^10–13^ while no association was observed in other four studies^6,9,14,15^. For that reason, in a recently published systematic review^16^ we sought to identify a signature of miRNAs of relevance in DLBCL. However, we observed that the studies performed usually considered a limited set of selected miRNAs, which limits the number of comparable results and centers the discussion on those miRNAs that are better known, leaving other miRNAs aside. Therefore, we considered that it was necessary to perform large-scale studies with a wider array of miRNAs using techniques such as next-generation sequencing that allow a deeper and unbiased identification of miRNAs with the potential to be used as biomarkers in DLBCL. Moreover, this technology allows the characterization of the whole miRNAome, providing an insight in the global mechanism of action of deregulated miRNAs.

Consequently, the main goals of the present study were to determine a miRNA set with utility in DLBCL diagnosis, classification, prognosis and treatment response, through the analysis of all the expressed miRNAs in DLBCL using small RNA sequencing, as well as to decipher the mechanism of action of deregulated miRNAs in the origin of the disease through the development of a miRNA-mRNA interaction network.

## Results

### MiRNAs deregulated in DLBCL

A total of 1584 miRNAs were identified through the miRNA sequencing analysis. A principal component analysis (PCA) was performed including the expression of all the miRNAs to obtain an overview of the grouping of samples according to their variance. Patient and control samples cluster as clearly separate groups (Supplementary Fig. S1). To obtain a comprehensive list of miRNAs deregulated in DLBCL, we compared the expression of each miRNA in 78 DLBCL samples at diagnosis with 17 non-tumoral ganglia from individuals without DLBCL. We noted that 146 miRNAs exhibited statistically significant differences in expression between samples of DLBCL patients at diagnosis and control samples. Of these miRNAs, 122 exhibited increased abundance in DLBCL, while 24 miRNAs exhibited decreased abundance. The 20 most discriminatory up- and down-regulated miRNAs are listed in Table 1.

**Table 1:** The 10 most upregulated and downregulated microRNAs in DLBCL patients vs. controls.

MicroRNA	Base Mean	log2FoldChange	p-value	padj
hsa-miR-210-3p	842.9	3.51	2.33 × 10^–29^	1.45 × 10^–26^
hsa-miR-944	60.9	4.10	2.19 × 10^–23^	6.80 × 10^–21^
hsa-miR-12136	76.05	26.94	3.64 × 10^–20^	6.47 × 10^–18^
hsa-miR-3681-5p	75.3	5.16	3.33 × 10^–20^	6.47 × 10^–18^
hsa-miR-378i	24.9	3.01	3.01 × 10^–17^	4.16 × 10^–15^
hsa-miR-4454	183.4	2.35	1.01 × 10^–16^	1.04 × 10^–14^
hsa-miR-1291	354.7	4.02	1.84 × 10^–16^	1.76 × 10^–14^
hsa-miR-7974	111.8	3.46	1.87 × 10^–15^	1.45 × 10^–13^
hsa-miR-183-5p	891.1	3.40	5.77 × 10^–15^	3.59 × 10^–13^
hsa-miR-146a-5p	33,085.5	2.11	2.05 × 10^–14^	1.16 × 10^–12^
hsa-miR-215-5p	53.8	− 4.42	6.74 × 10^–35^	8.39 × 10^–32^
hsa-miR-150-5p	6310.7	− 3.22	3.46 × 10^–25^	1.44 × 10^–22^
hsa-miR-224-5p	47.7	− 3.30	5.11 × 10^–21^	1.27 × 10^–18^
hsa-miR-194-5p	37.8	− 4.33	6.19 × 10^–17^	7.00 × 10^–15^
hsa-miR-452-3p	7.2	− 2.33	2.10 × 10^–16^	1.87 × 10^–14^
hsa-miR-335-5p	127.6	− 2.74	6.20 × 10^–16^	5.14 × 10^–14^
hsa-miR-145-5p	2060.5	− 2.16	2.65 × 10^–15^	1.94 × 10^–13^
hsa-miR-139-5p	48.8	− 2.26	5.40 × 10^–15^	3.57 × 10^–13^
hsa-miR-497-5p	332.5	− 2.07	3.53 × 10^–14^	1.91 × 10^–12^
hsa-miR-10a-3p	10.2	− 2.29	7.45 × 10^–12^	2.73 × 10^–10^

Positive Log2 Fold change represented the upregulation of microRNAs in DLBCL patients compared with healthy control individuals; Negative Log2 Fold change represented the downregulation of microRNAs in DLBCL patients compared with healthy control individuals; Base Mean: represented the average of the normalized count values, dividing by size factors, taken over all samples.

### MiRNAs differentially expressed among DLBCL subtypes

By analyzing expression profiling of 32 Germinal center B-cell like (GCB) and 21 Non-GCB DLBCL samples at diagnosis, eight miRNAs were found to be differentially expressed. Five of them were upregulated in GCB DLBCL patients (miR-129-2-3p, miR-4464, miR-3150b-3p, miR-138-5p and miR-129-5p) and three miRNAs were upregulated in Non-GCB subtype (miR-511-5p, miR-205-5p, miR-3652) (Table 2).

**Table 2:** MicroRNAs differentially expressed between GCB and Non-GCB DLBCL subtypes.

MicroRNA	Base Mean	log2FoldChange	p-value	Padj
hsa-miR-129-2-3p	4.9	4.12	1.50 × 10^–06^	0.00050
hsa-miR-4464	2.9	3.91	1.21 × 10^–05^	0.00151
hsa-miR-3150b-3p	49.0	2.00	1.90 × 10^–05^	0.00211
hsa-miR-138-5p	568.1	2.00	9.92 × 10^–05^	0.00661
hsa-miR-129-5p	18.2	2.90	0.00014	0.00891
hsa-miR-511-5p	7.6	− 2.01	9.92 × 10^–06^	0.00142
hsa-miR-205-5p	20.3	− 3.30	4.10 × 10^–05^	0.00342
hsa-miR-3652	8.9	− 2.44	0.00088	0.03137

Positive Log2 Fold change represented the upregulation of microRNAs in GCB DLBCL patients compared with Non-GCB individuals; and negative, upregulation in Non-GCB DLBCL; Negative Log2 Fold change represented the upregulation of microRNAs in Non-GCB DLBCL patients compared with GCB individuals; and negative, upregulation in Non-GCB DLBCL; Base Mean: represented the average of the normalized count values, dividing by size factors, taken over all samples.

### MiRNAs associated with DLBCL treatment response

Eleven miRNAs were differentially expressed between samples at diagnosis from patients with long-term complete remission (n = 50) and refractory patients (n = 12), of which, 10 miRNAs were upregulated in patients with complete remission (miR-12136, miR-129-5p, miR-129-1-3p, miR-3150b-3p, miR-127-3p, miR-3681-5p, miR-370-3p, miR-4464, miR-129-5p and miR-3928-3p) and one miRNA was downregulated in the same group of patients (miR-192-5p) (Table 3).

**Table 3:** MicroRNAs significantly associated with response to treatment.

MicroRNA	Base Mean	log2FoldChange	pvalue	padj
hsa-miR-12136	120.0	25.73	1.29 × 10^–13^	9.70 × 10^–11^
hsa-miR-129a-5p	42.7	5.09	2.86 × 10^–09^	1.08 × 10^–06^
hsa-miR-129-1-3p	7.4	4.08	1.86 × 10^–06^	0.00035
hsa-miR-3150b-3p	50.7	2.33	1.60 × 10^–05^	0.00241
hsa-miR-127-3p	3977.2	2.01	6.14 × 10^–05^	0.00661
hsa-miR-3681-5p	75.3	2.34	0.00016	0.01507
hsa-miR-370-3p	16.1	2.59	0.00041	0.02380
hsa-miR-4464	3.3	3.55	0.00117	0.04641
hsa-miR-129b-5p	27.5	2.88	0.00102	0.04641
hsa-miR-3928-3p	16.5	2.03	0.00113	0.04641
hsa-miR-192-5p	10,375.5	− 2.41	1.60 × 10–^07^	4.01 × 10^–05^

Positive Log2 Fold change represented the overexpression of microRNAs at diagnosis in patients with complete remission compared with refractory patients; and negative, downregulation in the same group of patients; Base Mean: represented the average of the normalized count values, dividing by size factors, taken over all samples.

### MiRNAs as prognostic biomarkers in DLBCL and their impact on survival

MiRNA expression at diagnosis of 50 patients with long-term remission and 16 patients that relapsed within 10 years after diagnosis was compared. The results revealed that seven miRNAs (miR-4444, miR-449c-5p, miR-3681-5p, miR-3928-3p, miR-449b-5p, miR-370-3p, and miR-4424) were significantly upregulated in patients with long-term remission and three miRNAs (miR-133a-3p, miR-208b-3p, and miR-205-5p) were significantly upregulated in patients that relapsed (Table 4).

**Table 4:** MicroRNAs significantly associated with prognosis.

MicroRNA	Base Mean	log2FoldChange	pvalue	padj
hsa-miR-4444	10.3	2.25	6.20 × 10^–05^	0.0089
hsa-miR-449c-5p	9.8	2.22	4.75 × 10^–05^	0.0089
hsa-miR-3681-5p	75.3	2.03	0.00021	0.014
hsa-miR-3928-3p	16.5	2.02	0.00018	0.014
hsa-miR-449b-5p	7.23	2.22	0.00036	0.021
hsa-miR-370-3p	16.1	2.12	0.00071	0.029
hsa-miR-4424	110.8	2.32	0.0010	0.041
hsa-miR-133a-2-3p	470.9	− 3.8	1.53 × 10^–06^	0.001
hsa-miR-133a-1-3p	69.29	− 2.52	6.78 × 10^–05^	0.0089
hsa-miR-208b-3p	12.94	− 3.08	0.00014	0.013
hsa-miR-205-5p	62.4	− 3.36	0.0002	0.014

Positive Log2 Fold change represented the overexpression of microRNAs in patients with long-term remission compared with relapsed patients; and negative, overexpression in relapsed patients. Base Mean: represented the average of the normalized count values, dividing by size factors, taken over all samples.

**Figure 1:**
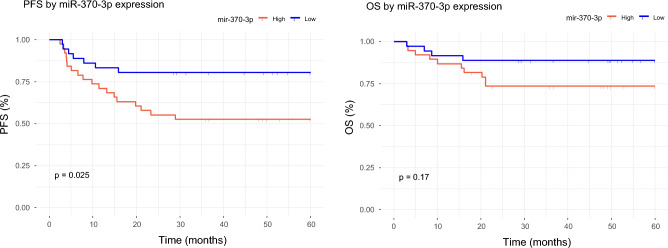
Survival analysis of miR-370-3p. The red line represents patients with high miR-370-3p expression and the blue line represents patients with low miR-370-3p expression.

Patient survival curves were generated to represent 5-year progression-free survival (PFS) and overall survival (OS), dividing the patients into high-expression and low-expression groups according to the mean expression of each of the ten miRNAs significantly associated with prognosis. The long-rank test revealed that, among the miRNAs associated with good prognosis, high expression of miR-370-3p was associated with better 5-year PFS (OS p-value = 0.17, and PFS p-value = 0.025) (Fig. 1). We also performed Cox PH multivariate analysis including two established indicators of DLBCL patient outcome (COO-Subtype and IPI) as covariates. However, the results of this analysis revealed that miR-370-3p was not associated with PFS independently of IPI and subtype.

### miRNA-mRNA interaction network analysis

For the 122 upregulated miRNAs found in our study (DLBCL *vs* controls), we identified a total of 482 experimentally validated targets using MirTarbase. Regarding the 24 downregulated miRNAs from our study, a total of 375 experimentally validated target genes were identified. Analysis of the GSE56315 dataset identified through the search in the GEO, revealed a total of 1918 significantly upregulated genes and 4545 downregulated genes in DLBCL patients compared with healthy control individuals.

**Figure 2:**
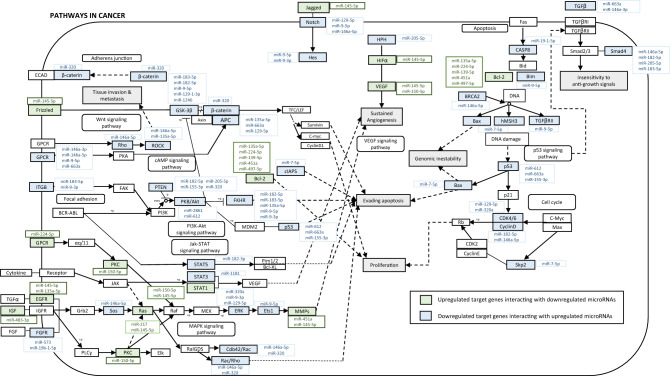
Genes of pathways in cancer targeted by downregulated and upregulated microRNAs (adapted from KEGG database).

Integration of both datasets showed that downregulated miRNAs targeted a total of 138 genes among those highly expressed in DLBCL (Supplementary Table S1), and upregulated miRNAs a total of 76 targets among those genes downregulated in DLBCL (Supplementary Table S2). Some miRNAs showed interactions with several targets (Supplementary Table S3). For instance, miR-9-5p was shown to target 25 genes downregulated in DLBCL, and miR-146a-5p and miR-182-5p were shown to target 24 and 20 genes downregulated in DLBCL, respectively. Some target genes showed interactions with several miRNAs (Supplementary Table S4). For instance, *FOXO1*, *BCL2*, *GS3KB* and *PTEN*, downregulated in DLBCL, are targeted by 7, 5, 5 and 4 miRNAs deregulated in DLBCL, respectively. In order to evaluate the pathways that could be affected by the deregulated miRNAs through their target genes, we performed a pathway enrichment analysis using the ConsensusPathDB web tool. Among the most significantly over-represented pathways, we identified some involved in cancer, such as FoxO signaling pathway, signaling by Receptor Tyrosine Kinases, and PI3K-Akt signaling pathway (Fig. 2).

## Discussion

In this study, we identified new signatures of miRNAs of relevance in DLBCL with potential to improve diagnosis, subtype characterization and treatment response through small RNA sequencing. To our knowledge, few reports exist in which miRNA sequencing were used to identify miRNA signatures in cancer, and only one which analyzed miRNAs with Next Generation Sequencing (NGS) in DLBCL^9^. Notably, deregulation of most of these miRNAs had not been reported previously in DLBCL.

Regarding miRNAs deregulated at diagnosis in DLBCL, 146 miRNAs differentially expressed between DLBCL samples and controls were identified, pointing to a relevant role of miRNAs in the pathogenesis of the disease. Of note, all miRNAs we previously proposed as a consistent signature of deregulation in DLBCL in a systematic review (miR-150-5p, miR-146a-5p, miR-155-5p and miR-21-5p)^16^ were identified in the current study following a similar expression pattern. Moreover, we also identified other miRNAs highly deregulated in DLBCL. Indeed, regarding the most upregulated and downregulated miRNAs, only miR-210-3p, miR-146a-5p, miR-129-5p, miR-215, miR-150-5p, miR-224-5p, miR-335-5p, miR-145-5p, miR-497-5p, and miR-151b had been studied previously in DLBCL.

Comparing miRNA expression in DLBCL GCB and Non-GCB subgroups, eight miRNAs were identified with subtype differentiation potential. Five of them were upregulated in GCB DLBCL patients (miR-129–2-3p, miR-4464, miR-3150b-3p, miR-138-5p and miR-129-5p) and three were upregulated in Non-GCB subtype (miR-511-5p, miR-205-5p, and miR-3652). Of note, miR-28-5p, which was proposed as a biomarker of GCB DLBCL in our previous systematic review, pending further confirmation^16^, was also differentially expressed among subtypes, although the logFC did not reach the threshold set for the present study. Interestingly, higher levels of miR-129-5p and miR-138-5p in the GCB subtype had been previously reported^6,9,14,17^. As part of the miRNA-mRNA interaction network analysis, we observed that they could target transcripts that are known to be deregulated in the formation of germinal center lymphomas^18^, including genes related with the cell cycle (*CDKN1A*), MAPK and NFkB signaling *(MAPK1).* Interestingly, we also found *TP53* as a target gene of miR-129-3p, miR-129-5p, and miR-138-5p. *TP53* gene is a crucial tumor suppressor that mediates cell-cycle arrest, DNA repair, apoptosis, senescence, and autophagy^19,20^, and its dysfunction is implicated in lymphomagenesis and disease progression. Recent studies have shown that *TP53* mutations have prognostic value in GCB-DLBCL, but not in Non-GCB-DLBCL^21^, which could suggest that GCB-DLBCL is *TP53*-dependent while Non-GCB DLBCL is *TP53*-independent, and thus, could support the role of miR-129-3p, miR-129-5p, and miR-138-5p in GBC subtype.

We also identified a predictive miRNA signature for therapy response, which included the upregulation of ten miRNAs (miR-12136, miR-129a-5p, miR-129-1-3p, miR-3150b-3p, miR-127-3p, miR-3681-5p, miR-370-3p, miR-4464, miR-129b-5p and miR-3928-3p) in association with good response and upregulation of miR-192-5p associated with chemoresistance to R-CHOP DLBCL treatment. Notably, deregulation profiling of most of these miRNAs in DLBCL had not been previously reported, with a limited number of studies carried out; but some of them, such as miR-192 had been studied in the context of other types of cancer^22,23^, supporting their role in drug response. Interestingly, miR-370-3p has been recently shown as a target of circ_0000877 and LINC00857 mediating their effect on DLBCL cells survival, which would be in agreement with its association with good response to therapy^24,25^. In addition to being putative biomarkers for R-CHOP treatment response prediction, these miRNAs could also be considered in the future as novel therapeutic targets for personalized miRNA-based therapy, which could be an alternative adjuvant therapy, although further studies are needed in this area of research before such therapies can be translated to the clinic.

In addition to treatment response, the ability to predict survival accurately may be crucial for initial treatment planning in patients with DLBCL. We identified seven miRNAs (miR-4444, miR-449c-5p, miR-3681-5p, miR-3928-3p, miR-449b-5p, miR-370-3p, miR-4424) significantly upregulated in patients with long-term remission and four miRNAs (miR-133a-3p, miR-133a-3p, miR-208b-3p, miR-205-5p) upregulated in relapsed patients. In addition, the survival analysis reveals that high expression of miR-370-3p is significantly associated with PFS. However, the association was not maintained when IPI and subtype were included in the model. As mentioned above, its role as a mediator of DLBCL cell survival in vitro has been stablished in two different studies. Thus, further studies are warranted to confirm its role as outcome predictor.

To gain insight into the global mechanism of action of deregulated miRNAs, we also performed a miRNA-mRNA interaction network analysis. Integration analysis determined that up to 214 genes could have altered expression in DLBCL because of miRNA deregulation. In this line, there were 138 genes with low expression in DLBCL that could be due, at least in part, to the increased expression of miRNAs identified in the current study. Moreover, 76 genes were highly expressed that could be due to a lack of regulation by the miRNAs lowly expressed here identified. Among the most interesting downregulated genes targeted by more than one miRNA overexpressed in DLBCL, we identified *FOXO1* and *PTEN,* involved in PI3K-Akt pathway^26^*,* predicted to be targeted by 5 (miR-182-5p, miR-183-5p, miR-135a-5p, miR-9-5p, and miR-9-3p) and 4 (miR-155, miR-320a, miR-205 and miR-182-5p) overexpressed miRNAs, respectively. *FOXO1* is a tumor suppressor and its downregulation leads to promoting tumorigenesis by favoring resistance to stress, proliferation, and increased cellular survival^27^. Abnormal cells that would normally undergo apoptosis may instead survive in the absence of *FOXO1,* which results in tumor expansion^28^. In fact, downregulation of *FOXO1* has been previously reported in DLBCLs^29^. In the same line, *PTEN* is a tumor suppressor that acts upstream of *FOXO1* negatively regulating *AKT.* Without PTEN regulation, activated AKT phosphorylates FOXO1, which is subsequently exported from the nucleus into the cytoplasm and degraded by the proteasome^30^. Interestingly, *PTEN* is the major negative regulator of the PI3K/AKT signaling pathway, which is constitutively activated in 25–50% of DLBCL according to recent studies^31^. Interestingly, miR-182-5p targeted both genes and could be a crucial miRNA modulating the PI3K/AKT pathway by targeting *PTEN,* upstream, and *FOXO1,* downstream. Further supporting our findings, a relation between miR-182 and miR-183 and *FOXO1* suppression have been previously described in classical Hodgkin lymphoma^32^.

Regarding the upregulated genes in DLBCL that could be a consequence of miRNA downregulation, the most significant result is *BCL2*. Evidence revealed that elevated expression of anti-apoptotic members such as *Bcl-2* is one of the major contributing factors to B cell lymphomagenesis^33^. Since then, many studies have determined that *BCL2* is one of the most important oncogenes in cancer and that it is linked with lymphoma development, particularly when c-MYC is overexpressed^34^. This gene could be regulated negatively by miR-135a-5p, miR-234-5p, miR-139-5p, miR-451a, and miR-497-5p. Therefore, the low expression of the mentioned miRNAs observed in DLBCL would contribute to BCL2 overexpression. In fact, previous studies have confirmed that deletion or downregulation of miRNAs is a mechanism for BCL2 overexpression^35^.

Finally, this study has some limitations that need to be addressed. First, we analyzed miRNA expression from formalin-fixed paraffin-embedded (FFPE) blocks, in which RNA quality is usually compromised. However, compared to mRNA, miRNAs are relatively resistant to RNase degradation^36^, and previous studies demonstrated that miRNA expression from FFPE samples is in good correlation with fresh frozen samples^37^. Secondly, patients were diagnosed in three different hospitals with different follow up routines, which could be a source of heterogeneity, although the treatment protocols were similar. Finally, we also have to mention that in this study the Han’s IHC algorithm was used to classify the patients into GCB and Non-GCB subtypes. Even though studies have shown clearly that the IHC-based classification provided very similar outcome prediction compared with the gene expression profiling based classification, which is the gold standard^38^, variable reproducibility of IHC stains and interpretations, may be the reason for inconsistent results^39^.

In conclusion, in the present study, a comprehensive analysis of miRNA profiles by next-generation sequencing technology allowed us to identify signatures that could be of relevance in DLBCL. Prospective studies are warranted to further validate our findings and explore new therapeutic options based on the miRNA profile of each patient. Moreover, we explored the mechanisms by which deregulated miRNAs contributes to DLBCL pathogenesis, with miR-182-5p playing a key role in PI3K/AKT pathway.

## Materials and methods

### Population of study

Samples from 78 DLBCL patients were obtained at diagnosis from formalin-fixed paraffin-embedded (FFPE) tumor tissue (50 patients with long-term complete remission, 12 patients with refractory disease, and 16 patients that relapsed within 10 years after diagnosis). Samples were collected from 1999 to 2018 at the Hematology Units of 3 Spanish reference hospitals (Cruces University Hospital, Donostia University Hospital, and Araba University Hospital). Control samples were obtained from non-tumoral ganglia from individuals without DLBCL, which were collected at Araba University Hospital. Demographic and clinical data were obtained from patient’s medical files by two independent clinical researchers (Table 5). Demographic information was also obtained for the controls. All patients were treated with R-CHOP or similar chemotherapy regimens, which included rituximab. The study was approved by the Clinical Research Ethical Committee of the Basque Country (P2016121). Signed informed consent was obtained from each participant and the study was carried out according to the Declaration of Helsinki.

**Table 5:** Demographic and clinical characteristics of DLBCL patients at diagnosis (n = 78).

Variables	Patients with DLBCL (n = 78)	Controls (n = 17)
Age		
Mean (range)	58.66 (21–81)	67.76 (31–86)
≥ 60	43	4
< 60	34	13
NA	1	0
Sex		
Male	41	9
Female	37	8
Stage		
I	7	
II	11	
III	23	
IV	27	
NA	10	
B symptoms		
Yes	31	
No	31	
NA	18	
IPI scores		
Low risk (0–1)	15	
Low to intermediate risk (2)	13	
Intermediate to high risk (3)	15	
High risk (4–5)	20	
NA	15	
Subtype IHC or molecular		
GCB	32	
Non-GCB	21	
NA	25	
Therapy		
Rituximab + standard chemotherapy	63	
Rituximab + intensified chemotherapy	15	
Therapy response		
CR	50	
Not CR	28	
LDH		
Normal	23	
Augmented	40	
NA	17	
β2-MG, β 2 microglobulin		
Normal	22	
Augmented	39	
NA	17	
5 years PFS		
Median, months	39.68	
5 years OS		
Median (range), months	46.63	

CR, complete response; DLBCL, diffuse large B-cell lymphoma; GCB, germinal center B cell; IHC, immunohistochemistry; IPI, International Prognostic Index; β2-MG, β 2 microglobulin; LDH, lactate dehydrogenase; PFS, progression free survival; OS, Overall survival; NA, not available.

### Sample processing, Small RNA-seq library preparation and sequencing

RNA was isolated from FFPE samples with the miRNeasy FFPE Kit (Qiagen, Hilden, Germany). One µg of total RNA was used for library preparation with TruSeq small RNA Sample Prep Kit (Illumina), according to the manufacturer's protocol. Libraries were analyzed using Agilent DNA High Sensitivity chip and sequenced Single Read, 50nts (v4) on Illumina’s HiSeq 2500 with a depth of approximately 10 million reads per sample. All the process was carried out at the Centre for Genomic Regulation (CRG). The data are openly available in GEO database, reference number GSE185796^40^.

### Bioinformatic analysis and differential miRNA expression

Reads were trimmed for the presence of the small RNA adapter using Skewer and filtered removing every sequence shorter than 15 and longer than 40 bases. The remaining ones were then aligned to the reference genome (GRCh38) using ShortStack and the resulting alignments used by htseq-count for determining the number of tags per gene. We used the annotation of the miRBase Sequence Database v22.1mirbase in order to select only miRNA genes. Every small RNA with less than 10 reads considering the sum of the reads in every condition was excluded, and we used the remaining genes for differential expression analysis with DESeq2. p-values were adjusted using False discovery rate (p-adj). Differentially expressed miRNAs were those with p-adj < 0.05 and a log_2_ fold change (logFC) > 2 or < − 2. Principal component analysis was performed using the prcomp package from R to analyze the transformed read counts of all miRNAs.

### Survival analysis

Survival analyses were conducted to confirm the absence of effect of changes in chemotherapy (standard vs. intensified) on survival. Additional survival analyses were performed to estimate the effect on the progression-free survival (PFS) and overall survival (OS) of those miRNAs differentially expressed between samples at diagnosis from patients with long-term remission and those that relapsed. The median value of the expression of those differentially expressed miRNAs was considered to categorize patients into low or high expression groups. Kaplan–Meier analysis was used to define the survival curves and the log-rank test was used to assess significance. For the multivariate analysis, we also considered the subtype and IPI status using the Cox proportional hazards (Cox PH) method. All calculations were performed using the Survival R package. Statistically significant associations with survival were those with p-value < 0.05.

### miRNA-mRNA interaction network

Using our own data on miRNA expression (DLBCL patients *vs* controls) and mRNA expression data contained in the Gene Expression Omnibus (GEO) database, we constructed a miRNA-mRNA interaction network to elucidate the mechanism of DLBCL development. First, for those miRNAs deregulated in patients compared to controls, target genes were identified using mirTarbase^41^ (http://mirtarbase.cuhk.edu.cn/php/index.php) to search for experimentally validated miRNA-mRNA interactions. For the current study, only strong miRNA-mRNA interactions were selected. Secondly, after a comprehensive search of databases in GEO (http://www.ncbi.nlm.nih.gov/geo/), the only microarray dataset (GSE56315) containing gene expression information of both DLBCL samples (n = 55) and healthy controls (n = 33) was downloaded^42,43^. Differentially expressed genes (DEG) were identified using GEO2R tool with default parameters. We established the following inclusion criteria for the DEGs: upregulated genes must have a logFC ≥ 2 and p-adj < 0.05, while downregulated genes must have a logFC ≤ − 2 and p-adj < 0.05. Finally, experimentally validated targets of differentially deregulated miRNAs were overlapped with mRNAs differentially expressed obtained from GEO databases as represented in Supplementary Fig. S2.

The intersection of the upregulated and downregulated genes was mapped using the Venn package (http://bioinformatics.psb.ugent.be/webtools/Venn/). Signaling pathway enrichment analysis (Consensus Pathway database http://cpdb.molgen.mpg.de/) were conducted with overlapping genes.

## Supplementary Information

Supplementary Information.

## Data Availability

The data are openly available in GEO database, reference number GSE185796.
