# Supplementary material for: microRNA sequencing for biomarker detection in the diagnosis, classification and prognosis of Diffuse Large B Cell Lymphoma
Source: Sci Rep. 2023 Jul 27;13:12159. doi: 10.1038/s41598-023-39271-7 (PMC10374576; doi:10.1038/s41598-023-39271-7)
Supplement: Supplementary file 1 — Supplementary Information. [file 41598_2023_39271_MOESM1_ESM.docx]

**microRNA sequencing for biomarker detection in the diagnosis, classification and prognosis of Diffuse Large B Cell Lymphoma**

Ane Larrabeiti-Etxebarria^1^, Nerea Bilbao-Aldaiturriaga^1,2,3^, Javier Arzuaga-Mendez^1,4^, Maialen Martin-Arruti^5^, Luca Cozzuto^6^, Ayman Gaafar^7^, Irune Ruiz-Diaz^5^, Isabel Guerra^8^, Idoia Martin-Guerrero^1,3^, Elixabet Lopez-Lopez^2,3*,^ Angela Gutierrez-Camino^3,9*^

^1^ Department of Genetics, Physical Anthropology and Animal Physiology, Faculty of Science and Technology, University of the Basque Country, UPV/EHU, Leioa, Spain

^2^ Department of Biochemistry and Molecular Biology, University of the Basque Country, UPV/EHU, Leioa, Spain

^3^ Pediatric Oncology Group, BioCruces Bizkaia Health Research Institute, Barakaldo, Spain

^4^ Hematologic Neoplasm Group, BioCruces Bizkaia Health Research Institute, Barakaldo

^5^ Pathology Department, Donostia University Hospital, San Sebastián, Spain

^6^ Centre for Genomic Regulation (CRG), The Barcelona Institute of Science and Technology, Barcelona, Spain

^7^ Pathology Department, Cruces University Hospital, Barakaldo, Spain

^8^ Pathology Department, Araba University Hospital, Vitoria, Spain

^9^ Division of Hematology-Oncology, CHU Sainte-Justine Research Center, Montreal, Canada

Corresponding author:

Elixabet Lopez-Lopez. Department of Biochemistry and Molecular Biology, Faculty of Science and Technology-University of the Basque Country (UPV/EHU) Barrio Sarriena s/n, 48940 Leioa, Spain. E-mail: elixabet.lopez@ehu.eus; Tel.: +34.946012951

^*^These authors contributed equally to this work.

**Supplementary Figure 1:** Graphical representation of principal component analysis of the expression of miRNAs among DLBCL patients and controls.

**Supplementary Figure 2:** Workflow of microRNA-mRNA network construction

**Supplementary table 1:** Upregulated miRNAs and downregulated target genes in DLBCL.

| *microRNA* | *Entrez-gene name* |
| --- | --- |
| hsa-miR-612  hsa-miR-2861 | *AKT2* |
| hsa-miR-135a-5p  hsa-miR-663a  hsa-miR-129-5p | *APC* |
| hsa-miR-7-5p  hsa-miR-182-5p  hsa-miR-205-5p  hsa-miR-135a-5p  hsa-miR-9-5p | *BCL2* |
| hsa-miR-182-5p  hsa-miR-146a-5p | *CCND2* |
| hsa-miR-129-5p  hsa-miR-320a | *CDK6* |
| hsa-miR-146a-5p  hsa-miR-146a-3p  hsa-miR-9-5p  hsa-miR-663a | *CXCR4* |
| hsa-miR-182-5p  hsa-miR-183-5p  hsa-miR-9-5p  hsa-miR-9-3p  hsa-miR-135a-5p | *FOXO1* |
| hsa-miR-183-5p  hsa-miR-182-5p  hsa-miR-9-5p  hsa-miR-129-1-3p  hsa-miR-1246 | *GSK3B* |
| hsa-miR-9-3p  hsa-miR-9-5p | *HES1* |
| hsa-miR-183-5p  hsa-miR-9-3p | *ITGB1* |
| hsa-miR-320a  hsa-miR-9-3p  hsa-miR-129-5p | *MAPK1* |
| hsa-miR-129-5p  hsa-miR-9-5p  hsa-miR-146a-5p | *NOTCH1* |

| *microRNA* | *Entrez-gene name* |
| --- | --- |
| hsa-miR-182-5p  hsa-miR-155-3p  hsa-miR-205-5p  hsa-miR-320a | *PTEN* |
| hsa-miR-320a  hsa-miR-146a-5p | *RAC1* |
| hsa-miR-146a-5p  hsa-miR-135a-5p | *ROCK1* |
| hsa-miR-146a-5p  hsa-miR-182-5p  hsa-miR-205-5p  hsa-miR-183-5p | *SMAD4* |
| hsa-miR-129-5p  hsa-miR-612 | *SP1* |
| hsa-miR-663a  hsa-miR-146a-5p | *TGFB1* |
| hsa-miR-612  hsa-miR-663a  hsa-miR-155-3p | *TP53* |
| hsa-miR-7-5p | *BAX* |
| hsa-miR-9-5p | *BCL2L11* |
| hsa-miR-146a-5p | *BRCA2* |
| hsa-miR-19b-1-5p | *CASP8* |
| hsa-miR-320a | *CTNNB1* |
| hsa-miR-205-5p | *EGLN2* |
| hsa-miR-9-5p | *ETS1* |
| hsa-miR-573 | *FGFR1* |
| hsa-miR-19b-1-5p | *FGFR2* |
| hsa-miR-18a-3p | *KRAS* |
| hsa-miR-7-5p | *MSH3* |
| hsa-miR-146a-5p | *RHOA* |
| hsa-miR-7-5p | *SKP2* |
| hsa-miR-146a-5p | *SOS1* |
| hsa-miR-146a-5p | *STAT1* |
| hsa-miR-1181 | *STAT3* |
| hsa-miR-9-5p | *TGFBR2* |
| hsa-miR-182-3p | *STAT5B* |
| hsa-miR-7-5p | *XIAP* |

**Supplementary table 2**: Downregulated microRNAs and upregulated target genes in DLBCL.

| *microRNA* | *Entrez-gene name* |
| --- | --- |
| hsa-miR-145-5p | *EGFR* |
| hsa-miR-135a-5p | *EGFR* |
| hsa-miR-217 | *KRAS* |
| hsa-miR-224-5p | *KRAS* |
| hsa-miR-483-3p | *SMAD4* |
| hsa-miR-224-5p | *SMAD4* |
| hsa-miR-145-5p | *STAT1* |
| hsa-miR-150-5p | *STAT1* |
| hsa-miR-150-5p | *VEGFA* |
| hsa-miR-145-5p | *VEGFA* |
| hsa-miR-135a-5p | *APC* |
| hsa-miR-224-5p | *EDNRA* |
| hsa-miR-145-5p | *EPAS1* |
| hsa-miR-145-5p | *FZD7* |
| hsa-miR-483-3p | *IGF1* |
| hsa-miR-145-5p | *JAG1* |
| hsa-miR-145-5p | *MMP1* |
| hsa-miR-451a | *MMP2* |
| hsa-miR-451a | *MMP9* |
| hsa-miR-150-5p | *PRKCA* |

**Supplementary Table 3:** Downregulated target genes showing interactions with several upregulated miRNAs

| Target genes | microRNA |
| --- | --- |
| AKT2 | hsa-miR-612; hsa-miR-2861 |
| ALDH5A1 | hsa-miR-210-3p; hsa-miR-147b |
| APC | hsa-miR-135a-5p; hsa-miR-663a; hsa-miR-129-5p |
| BCL2 | hsa-miR-7-5p; hsa-miR-182-5p; hsa-miR-205-5p; hsa-miR-135a-5p; hsa-miR-9-5p |
| CCND2 | hsa-miR-182-5p; hsa-miR-146a-5p |
| CDK6 | hsa-miR-129-5p; hsa-miR-320a |
| CREB1 | hsa-miR-182-5p; hsa-miR-9-5p |
| CXCR4 | hsa-miR-146a-5p; hsa-miR-9-5p; hsa-miR-663a; hsa-miR-146a-3p |
| ELAVL1 | hsa-miR-9-5p; hsa-miR-146a-5p |
| EZR | hsa-miR-183-5p; hsa-miR-205-5p |
| FBXW7 | hsa-miR-182-5p; hsa-miR-182-3p; hsa-miR-155-3p |
| FOXO1 | hsa-miR-182-5p; hsa-miR-183-5p; hsa-miR-183-5p; hsa-miR-9-5p; hsa-miR-9-3p; hsa-miR-135a-5p; hsa-miR-135a-5p |
| FOXO3 | hsa-miR-182-5p; hsa-miR-9-5p |
| GSK3B | hsa-miR-183-5p; hsa-miR-182-5p; hsa-miR-9-5p; hsa-miR-129-1-3p; hsa-miR-1246 |
| HES1 | hsa-miR-9-3p; hsa-miR-9-5p |
| HIF3A | hsa-miR-210-3p; hsa-miR-147a |
| IGF2BP3 | hsa-miR-9-5p; hsa-miR-129-5p |
| ITGB1 | hsa-miR-183-5p; hsa-miR-9-3p |
| MAPK1 | hsa-miR-320a; hsa-miR-9-3p; hsa-miR-129-5p |
| MTSS1 | hsa-miR-182-5p; hsa-miR-135a-5p |
| NOTCH1 | hsa-miR-129-5p; hsa-miR-9-5p; hsa-miR-146a-5p |
| PTEN | hsa-miR-182-5p; hsa-miR-155-3p; hsa-miR-205-5p; hsa-miR-320a |
| RAC1 | hsa-miR-320a; hsa-miR-146a-5p |
| RECK | hsa-miR-182-5p; hsa-miR-183-5p |
| ROCK1 | hsa-miR-146a-5p; hsa-miR-135a-5p |
| RUNX2 | hsa-miR-205-5p; hsa-miR-320a; hsa-miR-135a-5p |
| SIAH1 | hsa-miR-135a-5p; hsa-miR-944 |
| SMAD4 | hsa-miR-146a-5p; hsa-miR-182-5p; hsa-miR-205-5p; hsa-miR-183-5p |
| SOX2 | hsa-miR-1181; hsa-miR-146a-5p |
| SP1 | hsa-miR-129-5p; hsa-miR-612 |
| TGFB1 | hsa-miR-663a; hsa-miR-146a-5p |
| TP53 | hsa-miR-612; hsa-miR-663a; hsa-miR-155-3p |
| YY1 | hsa-miR-7-5p; hsa-miR-205-5p |

**Supplementary Table 4:** Upregulated target genes showing interactions with several down regulated miRNAs

| Target genes | microRNA |
| --- | --- |
| CD44 | hsa-miR-216a-5p; hsa-miR-145-5p |
| CDH2 | hsa-miR-194-5p; hsa-miR-145-5p |
| CEBPD | hsa-miR-95-3p; hsa-miR-135a-5p |
| EGFR | hsa-miR-145-5p; hsa-miR-135a-5p |
| KLF4 | hsa-miR-145-5p; hsa-miR-135a-5p |
| KRAS | hsa-miR-217; hsa-miR-224-5p |
| MTDH | hsa-miR-145-5p; hsa-miR-217 |
| SMAD4 | hsa-miR-483-3p; hsa-miR-224-5p |
| STAT1 | hsa-miR-145-5p; hsa-miR-150-5p |
| VEGFA | hsa-miR-145-5p; hsa-miR-150-5p |
| ZEB2 | hsa-miR-215-5p; hsa-miR-335-5p |
